# Anti-Steatotic Effects of *Chlorella vulgaris*, *Nannochloropsis gaditana* and *Gracilaria vermiculophylla* Algae Extracts in AML-12 Hepatocytes

**DOI:** 10.3390/nu15081960

**Published:** 2023-04-19

**Authors:** Maitane González-Arceo, Jenifer Trepiana, Leixuri Aguirre, Jone Ibarruri, Marta Martínez-Sanz, Marta Cebrián, Isidra Recio, María P. Portillo, Saioa Gómez-Zorita

**Affiliations:** 1 Nutrition and Obesity Group, Department of Nutrition and Food Science, University of the Basque Country (UPV/EHU) and Lucio Lascaray Research Institute, 01006 Vitoria-Gasteiz, Spain; maitane.gonzalez@ehu.eus (M.G.-A.);; 2 CIBEROBN Physiopathology of Obesity and Nutrition, Institute of Health Carlos III, 01006 Vitoria-Gasteiz, Spain; 3 Bioaraba Health Research Institute, 01006 Vitoria-Gasteiz, Spain; 4 AZTI, Food Research, Basque Research and Technology Alliance (BRTA), Parque Tecnológico de Bizkaia, Astondo Bidea, 609, 48160 Derio, Spain; 5 Instituto de Investigación en Ciencias de la Alimentación, CIAL (CSIC-UAM), Nicolás Cabrera, 9, 28049 Madrid, Spain

**Keywords:** microalgae, macroalgae, seaweed, extracts, non-alcoholic fatty liver disease, steatosis, proteins, lipid metabolism

## Abstract

Non-alcoholic fatty liver disease (NAFLD) is considered the most common chronic liver alteration whose prevalence is increasing in Western countries. Microalgae and macroalgae have attracted great interest due to the high content in bioactive compounds with beneficial effects on health. The aim of the present study is to assess the potential interest of extracts rich in proteins obtained from the microalgae *Chlorella vulgaris* and *Nannochloropsis gaditana* and the macroalga *Gracilaria vermiculophylla* in the prevention of lipid accumulation in AML-12 hepatocytes. Toxicity was not observed at any of the tested doses. Both microalgae and the macroalga were effective in preventing triglyceride accumulation, with *Nannochloropsis gaditana* being the most effective one. Although the three algae extracts were able to increase different catabolic pathways involved in triglyceride metabolism, the mechanisms underlying the anti-steatotic effect were different in each algae extract. In conclusion, the present study demonstrates that *Chlorella vulgaris*, *Nannochloropsis gaditana* and *Gracilaria vermiculophylla* extracts are able to partially prevent the accumulation of triglycerides induced by palmitic acid in cultured hepatocytes, a model used to mimic the steatosis induced in liver by dietary patterns rich in saturated fat.

## 1. Introduction

Non-alcoholic fatty liver disease (NAFLD), characterised by the excess of lipid accumulation within hepatocytes (>5%) [1], is the most common chronic liver disease in Western countries, affecting approximately 25% of the adult population [2]. Its prevalence continues to grow due to the increase in sedentary behaviours and westernisation of the diet, factors that contribute to the above-mentioned metabolic alteration. The development of NAFLD is closely related to obesity and insulin resistance [3]. Recently, a panel of experts has proposed renaming NAFLD as metabolic dysfunction-associated fatty liver disease (MAFLD) since it more accurately reflects the heterogeneous pathogenesis and the underlying metabolic abnormalities [4].

Nowadays, there is no pharmacological treatment approved for liver steatosis. Diet and physical activity are the main common strategies used to prevent or treat this hepatic alteration [5]. Consequently, there is a growing interest in the development of new therapies [2] based, for instance, on bioactive molecules present in foodstuffs and plants. In this context, many authors are focusing their attention on marine algae, which contribute to the sustainable blue growth in the European Union, a strategy that ranges from responsible food systems to decarbonisation, as well as biodiversity and coastal resilience to circularity [6]. Algae are rich in several bioactive compounds such as pigments, polyunsaturated fatty acids, phenolic compounds, peptides, lipids, vitamins, polysaccharides or sterols, among others. It has been reported that they exert antioxidant, anti-inflammatory, anti-obesity, anti-diabetic, hypolipidemic, anti-hypertensive and anti-cancer activities [7,8,9]. As a result, diets supplemented with these organisms, both micro- and macroalgae, may have positive effects on chronic diseases [10,11,12,13].

Regarding the effects of algae on hepatic steatosis, although several species of both microalgae and macroalgae have demonstrated to have a positive effect on this alteration, the reported studies are still scarce [10]. The aim of the present study is to assess the potential interest of extracts obtained from the microalgae *Chlorella vulgaris* and *Nannochloropsis gaditana* and from the macroalga *Gracilaria vermiculophylla* in the prevention of liver steatosis. To our knowledge, the activity of these algae on hepatic triglyceride accumulation has yet to be studied. Furthermore, our aim is to identify the mechanisms responsible for this effect. 

## 2. Materials and Methods

### 2.1. Algae Extract Preparation

*Gracilaria vermiculophylla* was manually collected in September 2019 in the Bidasoa estuary (Hondarribia, Gipuzkoa, Spain). After collection and washing with marine water in the field, a second washing with fresh water was carried out in the lab to remove the impurities. After, samples were vacuum packed and frozen at −20 °C. Both microalgae (*Chlorella vulgaris* and *Nannochloropsis gaditana*) were provided by NEOALGAE (Gijón, Asturias, Spain) in frozen fresh-paste format (20–25% total solids) and stored at −20 °C until use. Before extraction, frozen biomass was thawed at 40 °C and diluted to the desired solid content with distilled water. 

Microalgae extracts were produced by adapting the methodology of Safi et al. [14,15]. A suspension of microalgae at 10% dry weight and adjusted to pH 12 with NaOH 10 M was prepared. The suspension was vacuum percolated through 100 μm filters to avoid issues with the Ultra High-Pressure Homogenizer (UHPH) equipment MicroDeBee (Bee International, South Easton, MA, USA) and stored in cold and dark atmosphere to avoid deterioration. The applied UHPH conditions were 250 MPa, 250 µm orifice and three cycles. The samples were submerged in an ice-water bath for proper preservation. After the UHPH process, samples were centrifuged at 10,000× *g* for 10 min at 4 °C (avoiding direct light). Soluble proteins were collected in the supernatant. These proteins were precipitated by adjusting the pH to 3.0 with 6 M HCl. Samples were again centrifuged at 10,000× *g* for 10 min at 4 °C, and the pellet was resuspended in 0.1 M phosphate buffer with a pH of 7.5. 

Macroalga extract was prepared as follows: frozen samples were thawed in a water bath at 40 °C and crushed to a particle size between 1 and 5 mm. A suspension of the macroalga in water at 5% of dry weight solids was prepared, grinded with a homogeniser (Ultra-turrax-IKA-T25, Staufen, Germany) for two minutes at 18,000 rpm and adjusted to a pH of 12 with NaOH 10 M. The suspension was then treated by ultrasounds (VibraCell 75042, Bioblock Scientific, Illkirch, France) for 1 h and 30 min, in cycles of 59 s ON and 15 s OFF. This treatment was also submerged in an ice-water bath to avoid an increase in temperature and protected from light to prevent alterations in bioactive compounds. After the extraction process, samples were handled in the same way as the microalgae suspensions after the UHPH procedure.

### 2.2. Algae Extract Composition

The proximate composition of the extracts was analysed according to the Association of Official Analytical Chemists (AOAC) Official Methods [16]. The moisture of the samples was determined through a drying process at 100 °C to a constant weight (method 934.01). Crude protein content was determined by Kjeldahl methodology, using a conversion factor for nitrogen-to-protein of N × 6.25 and 4.59 for microalgae and macroalga, respectively (method 955.04). Crude fat content was determined by Soxhlet methodology (method 920.39). Carbohydrate content was calculated by the difference in 100 g of algae extract. The total phenolic content was measured following the procedure described by Singleton and Rossi [17]. 

### 2.3. Characterization of the Protein Fraction

Sodium dodecyl sulphate-polyacrylamide gel electrophoresis (SDS-PAGE) was used on the algae extracts. Based on the protein content estimated by Kjeldahl, the extracts were dissolved in sample buffer, which contained Tris-HCl (0.05 M, pH 6.8, SDS (1.6% *w*:*v*), glycerol (8% *v*:*v*), β-mercaptoethanol (2% *v*:*v*) and bromophenol blue indicator (0.002% *w*:*v*) at protein concentrations of 0.8, 1.0 and 1.5 mg/mL. The samples were heated at 95 °C for 5 min and were subsequently loaded on the 12% Bis-Tris polyacrylamide gel (Criterion XT, Bio-Rad, Richmond, CA, USA). Electrophoretic separations were carried out at 150 V using XT-MES as a running buffer (Bio-Rad) in the Criterion cell (Bio-Rad). The gels were stained with Coomassie Blue (Instant Blue, Expedeon, Swavesey, UK), and images were taken with a Molecular Imager VersaDoc™ MP 5000 system (Bio-Rad, Hercules, CA, USA).

The protein/peptide size distribution was evaluated with high-performance size-exclusion chromatography (HPLC-SEC) using an ultra-high performance liquid chromatography instrument (Waters) equipped with a bioZenTM 1.8 µm 150 × 4.6 mm column and a bioZenTM SEC-2, 4.6 mm pre-column (Phenomenex). The mobile phase was a 55:45:0.1 water:acetonitrile:TFA (*v*/*v*/*v*) solution with a flow of 100 µL/min. The algae extracts were dissolved in water at a concentration of 3 mg/mL, diluted in the mobile phase to obtain a protein concentration of 1.5 mg/mL and centrifuged for 5 min at 12,800× *g* at room temperature to separate the soluble fraction. A total of 3 μL of each sample was injected into the system and the absorbance was registered at wavelengths of 214 and 280 nm. Peptides of known molecular weight (insulin with 5800 Da and peptides VPFPGPI and VYII with 888 Da and 506 Da, respectively) were used as molecular weight markers using the same chromatographic conditions.

### 2.4. Cell Culture and Treatment

A mouse hepatocyte AML12 cell line (alpha mouse liver 12; ATCC^®^ CRL-2254™), obtained from the American Type Culture Collection (ATCC, Manassas, VA, USA), was maintained in 75 cm^2^ flasks in DMEM/HAM’s F12 Glutamax and supplemented with 10% heat inactivated foetal bovine serum, 5 μg/mL insulin, 5 μg/mL transferrin, 5 ng/mL selenium, 40 ng/mL dexamethasone and 1% penicillin/streptomycin (10,000 U/mL) at standard cell culture conditions (37 °C, 5% CO_2_). When the cell monolayer reached 75% of confluence, cells were detached with a solution of trypsin-EDTA and harvested to perform subsequent experiments.

AML12 cells were incubated with palmitic acid (PA) to create an in vitro model of steatotic hepatocytes [18]. Cells were briefly exposed or not to 0.3 mM of PA to induce triglyceride accumulation and co-incubated with algae extracts for 18 h, adjusting the final concentration of each algae extract in culture media to 10, 25, 50 or 150 μg/mL. The control groups received the same amount of the vehicle. Each experiment was performed at least three times.

After 18 h, the incubation medium was collected, and cells were harvested according to the subsequent analysis. Cells used for triglyceride determination and protein immunodetection were collected in 10 mM Tris-HCl pH 7.4, 150 mM NaCl and 1 mM ethylenediaminetetraacetic acid (EDTA) buffer through scrapping. The cell suspension was then sonicated with 5 s bursts in a Branson Sonifier SFX550 (San Luis, MO, USA) fitted with a microtip. Cells used for RNA extraction were collected in TRIzol^®^ reagent. 

### 2.5. Cell Viability Assay

Cell viability was assessed using the crystal violet assay, based on cell staining with crystal violet [19]. Following treatment, AML12 cells were briefly washed with phosphate buffered saline (PBS), fixed in 3.7% formaldehyde and stained with 0.25% crystal violet in the dark for 20 min. Lastly, the resulting crystals were solubilised with 33% acetic acid, and the absorbance was registered at 590 nm in an iMark microplate reader (Bio-Rad, Hercules, CA, USA). Cell viability was expressed as the percentage of the control cells.

### 2.6. Determination of Triglyceride Content

Triglyceride content in cell suspension was measured using a commercial kit (Spinreact, Girona, Spain). Protein measurements were performed using the Bradford method [20]. Triglyceride content values were obtained as mg triglycerides/mg protein and expressed as the percentage of the control cells.

### 2.7. Detection of Alanine Aminotransferase (ALT/GPT) Levels in Cell Culture Medium

To determine ALT/GPT levels in cell culture medium, a commercial kit (BioSystems, Barcelona, Spain) was used. 

### 2.8. Analysis of Gene Expression by Real-Time PCR

RNA was extracted from cells using RNeasy Mini Spin Columns (Qiagen, Carlsbad, CA, USA) according to the manufacturer’s instructions. After DNase treatment (Ambion, Foster City, CA, USA), the integrity of the RNA was verified and quantified using an RNA 6000 Nano Assay (Thermo Scientific, Wilmington, DE, USA). A total of 1.5 μg of total RNA in a total reaction volume of 30 μL from each sample was reverse-transcribed into complementary DNA (cDNA) using an iScript cDNA Synthesis Kit (Bio-Rad, Hercules, CA, USA). Reactions were incubated initially at 25 °C for 5 min, subsequently at 42 °C for 30 min and finally at 85 °C for 5 min.

Adipose triglyceride lipase (*Atgl*), carnitine palmitoyltransferase I a (*Cpt1a*), citrate synthase (*Cs*), fatty acid synthase (*Fasn*), long chain acyl-CoA dehydrogenase (*Acadl*), mitochondrial transcription factor A (*Tfam*) and uncoupling protein 2 (*Ucp2*) mRNA levels were quantified. Beta actin (*Actb*) served as housekeeping for posterior normalisation. An aliquot of 4.75 μL of diluted cDNA sample was amplified by Real-Time PCR in a 12.5 μL reaction volume. The cDNA samples were amplified in an iCycler-MyiQ Real-Time PCR Detection System (Bio-Rad, Hercules, CA, USA) in the presence of SYBR Green Master Mix (Applied Biosystems, Foster City, CA, USA) and the sense and antisense primers (300 nM each except for *Fas*, where a concentration of 600 nM was used). The primer sequences are described in Table 1. The PCR parameters were as follows: initial 2 min at 50 °C, denaturation at 95 °C for 10 min, followed by 40 cycles of denaturation at 95 °C for 15 s, annealing at 60 °C for 30 s and extension at 60 °C for 30 s. For *Acadl* and *Fasn*, the annealing temperature was 61 and 62.2 °C, respectively. 

Acetyl-CoA carboxylase (*Acc*) and diacylglycerol acyltransferase (*Dgat2*) were amplified using TaqMan probes. *Actb* mRNA levels were similarly measured and served as the reference gene. In total, 4.5 μL of each diluted cDNA sample was added to the PCR reagent mixture (final volume of 10 μL), which consisted of TaqMan Fast Advanced Master Mix (Applied Biosystems, Vilna, Lithuania) and TaqMan Gene Expression Assay Mix (Applied Biosystems, Foster City, Ca, USA) containing specific primers and probes (Table 1). The PCR parameters were as follows: initial 2 min at 50 °C, denaturation at 95 °C for 2 min, followed by 40 cycles of denaturation at 95 °C for 3 s and combined annealing and extension at 60 °C for 30 s.

In all cases, the results were expressed as fold changes of the threshold cycle (Ct) value relative to controls using the 2^−ΔΔCt^ method [21].

### 2.9. Analysis of Protein Expression by Western Blot

Solute carrier family 27 member 2 (FATP2) and microsomal triglyceride transfer protein (MTTP) were assessed by western blot. The protein concentration was determined according to the Bradford protocol [20]. Protein samples (40 μg) were denaturalised at 95 °C for 3 min in Laemmli buffer [22] and loaded into 4–15% Mini-PROTEAN TGX Precast Gels (BioRad, Hercules, CA, USA). The proteins were then transferred onto PVDF membranes (Millipore, MA, USA) by electroblotting and later blocked with 5% casein and 0.5% bovine serum albumin (BSA) PBS-tween buffer for 2 h at room temperature. Subsequently, membranes were incubated with anti-FATP2 (1:500) (Santa Cruz Biotech, Santa Cruz, CA, USA), anti-MTTP (1:500) (Abcam, Cambridge, UK) and anti-α-tubulin (1:2000) (Cell Signaling, Beverly, MA, USA) for at least 1 h at room temperature and kept at 4 °C overnight. After washing, membranes were incubated with secondary antibodies (mouse anti-goat IgG; 1:5000 and mouse anti-rabbit IgG; 1:5000) (Santa Cruz Biotech, Santa Cruz, CA, USA); the immunoreactive proteins were detected by the Forte Western HRP substrate (Millipore; Burlington, MA, USA), and the blots were imaged by scanning with the ChemiDoc™MP Imaging System (Bio-Rad, Hercules, CA, USA). α-tubulin was used as housekeeping.

### 2.10. Statistical Analysis

The results are presented as mean ± standard error of the mean (SEM). Statistical analysis was performed using SPSS 26.0 (SPSS, Chicago, IL, USA). The normal distribution of the data was tested using the Shapiro–Wilk test. Data from the groups treated with algae extracts were compared with control cells or with PA cells using the Student’s *t* test or Mann–Whitney’s U test as appropriate. Statistical significance was established at the *p* < 0.05 level.

## 3. Results

### 3.1. Composition of Algae Extracts

The proximal composition of algae extracts is shown in Table 2. Dry weights were 90.27 ± 0.80 for *Chlorella vulgaris*, 90.02 ± 0.13 for *Nannochloropsis gaditana* and 91.70 ± 2.38 for *Gracilaria vermiculophylla*. The lowest fat percentage was found in *Gracilaria vermiculophylla* extract, and the highest one was found in the *Chlorella vulgaris* extract. By contrast, the percentage of protein was quite similar in the three extracts. Finally, regarding minerals, the *Nannochloropsis gaditana* extract showed a two-fold percentage compared to the other two extracts. 

The *Chlorella vulgaris* extract contained 12.27 ± 0.39 mg of total polyphenols/dry weight, calculated as gallic acid equivalents, *Nannochloropsis gaditana* 9.08 ± 1.56 mg and *Gracilaria vermiculophylla* 2.72 ± 0.08 mg. Thus, the extract of *Chlorella vulgaris* had the highest concentration and *Gracilaria vermiculophylla* had the lowest. 

**Figure 1:**
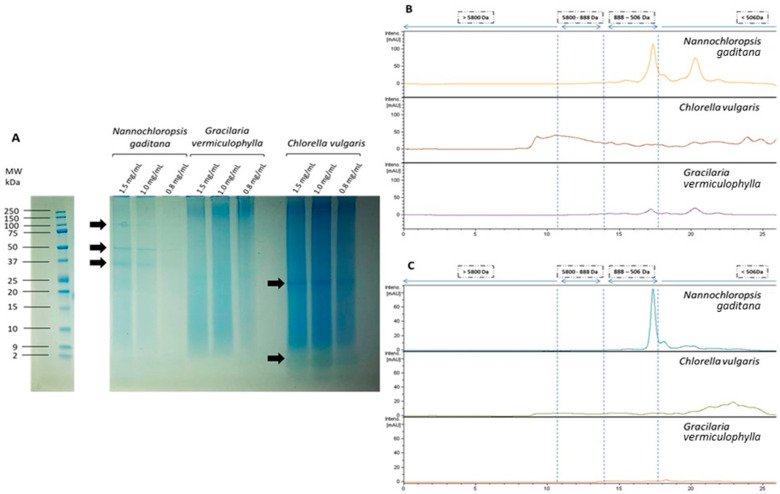
(**A**) SDS-PAGE protein profiles of the algae extracts at different protein concentrations. The arrows point towards the most intense bands. (**B**,**C**) HPLC-SEC chromatograms of the algae extracts detected at wavelengths of 214 nm (**B**) and 280 nm (**C**). The chromatograms for the three samples are shown at the same intensity scale.

The molecular weight (MW) distribution of proteins and peptides (above 10 kDa) in the algae extracts was studied through SDS-PAGE under denaturising conditions. As shown in Figure 1A, while some bands were clearly visible in the two microalgae extracts, it was not possible to identify protein bands in the case of the *Gracilaria* macroalga, even at the highest protein concentration tested. This was most likely caused by the lower solubility of this extract and its higher polysaccharide concentration, thus, hindering the transport and staining of the protein through the well. Regarding the *Chlorella* microalga, the most intense band was detected within the range of 20–25 kDa. Moreover, a coloured band lower than 2 kDa was also detected. In the case of the *Nannochloropsis* microalga, the main bands corresponded to 37 kDa, 50 kDa and 100 kDa. 

The molecular weight distribution of the extracts was further studied by means of HPLC-SEC, and representative chromatograms obtained at wavelengths of 214 nm and 280 nm (specific for amino acid residues with aromatic rings) are shown in Figure 1B,C, respectively. With regards to the *Chlorella vulgaris* extract, the chromatogram at 214 nm showed a very heterogeneous size distribution, with peaks appearing in a wide range of MW, while at 280 nm, only small peptides (<500 Da) were detected. The extract from *Nannochloropsis gaditana* revealed more intense peaks, specifically within the range of 1–0.5 kDa and <0.5 kDa. Contrarily, the extract from the macroalga *Gracilaria* showed a very low intensity, once again suggesting the low solubility of the proteins from this extract. Thus, only two small peaks corresponding to low MW compounds (<1 kDa) were visible in the chromatograms.

### 3.2. Cell Viability

**Figure 2:**
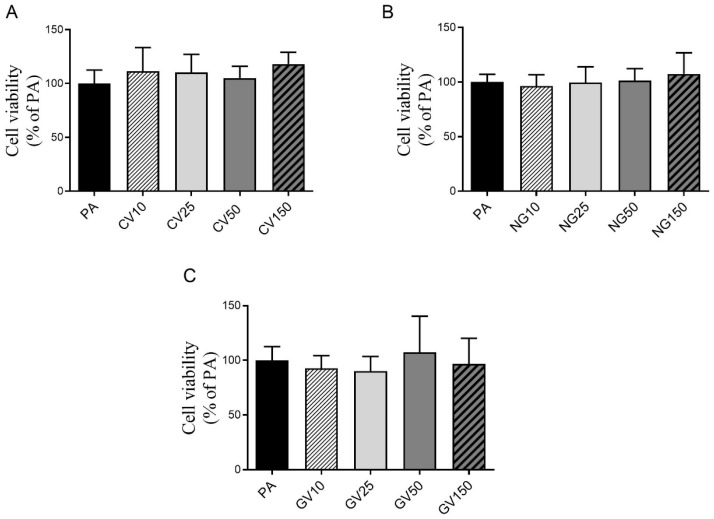
Cell viability in AML12 hepatocytes exposed to 0.3 mM of palmitic acid (PA) with or without *Chlorella vulgaris* (CV) (**A**), *Nannochloropsis gaditana* (NG) (**B**) and *Gracilaria vermiculophylla* (GV) (**C**) at 10, 25, 50 or 150 μg/mL for 18 h. Data are means ± SEM (standard error of the mean).

Hepatocytes incubated with 10, 25, 50 or 150 μg/mL of each algae extract, together with PA, showed no loss of viability compared with cells incubated with PA alone (Figure 2A–C). However, cell viability was decreased by 36% in the PA group with regards to the control group. 

### 3.3. Effects on Triglyceride Accumulation

**Figure 3:**
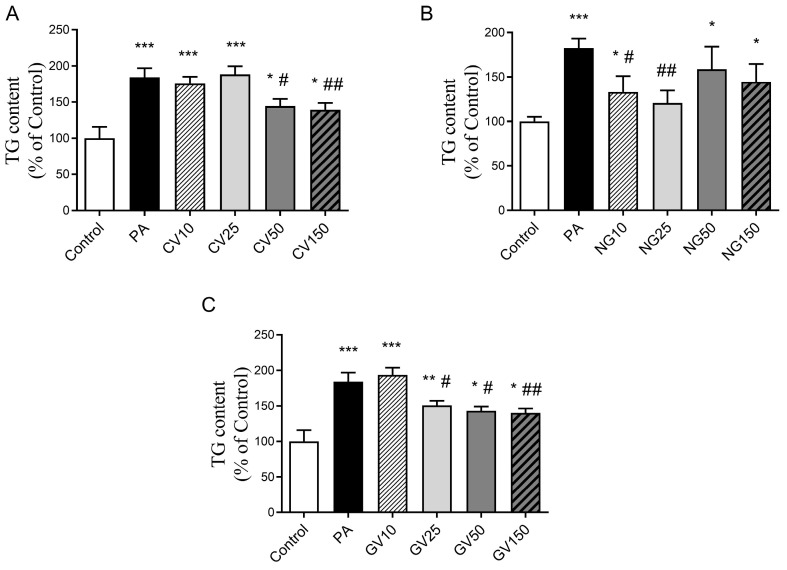
Triglyceride (TG) content in AML12 hepatocytes exposed or not to 0.3 mM of palmitic acid (PA) with or without *Chlorella vulgaris* (CV) (**A**), *Nannochloropsis gaditana* (NG) (**B**), and *Gracilaria vermiculophylla* (GV) (**C**) at 10, 25, 50 or 150 μg/mL for 18 h. Data are means ± SEM (standard error of the mean). * *p* < 0.05, ** *p* < 0.01 and *** *p* < 0.001 vs. control cells, # *p* < 0.05 and ## *p* < 0.01 vs. PA cells.

When cells were treated with *Chlorella vulgaris*, only those receiving 50 or 150 μg/mL of the microalga extract showed a decrease in triglyceride content (−22% and −24%, respectively) (Figure 3A). *Nannochloropsis gaditana* extract prevented triglyceride accumulation when cells were exposed to 10 or 25 μg/mL (−27% and −34%, respectively), but not at higher doses (Figure 3B). After treatment with *Gracilaria vermiculophylla*, the dose of 10 μg/mL proved ineffective, whereas the other three doses significantly avoided triglyceride accumulation induced by PA (−18%, −22% and −24%, respectively) (Figure 3C). In all cases, with the exception of *Nannochloropsis gaditana* at 25 μg/mL, the prevention of triglyceride accumulation was partial because values did not reach those found in the control hepatocytes.

### 3.4. Detection of ALT/GPT Level in Cell Culture Medium

The ALT/GPT concentration was measured in cell culture medium after the treatment of hepatocytes, with the dose of each algae extract showing the greatest reduction in triglyceride content: that is, 150 μg/mL for *Chlorella vulgaris* and *Gracilaria vermiculophylla* and 25 μg/mL for *Nannochloropsis gaditana*. 

**Figure 4:**
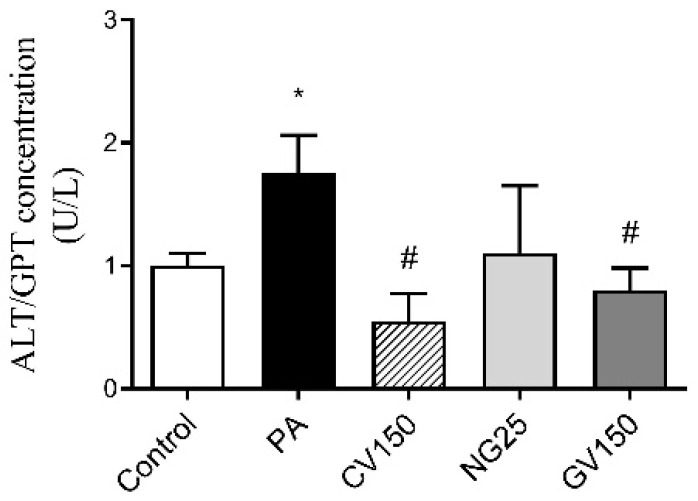
Alanine aminotransferase (ALT/GPT) level in cell culture medium of AML12 hepatocytes exposed or not to 0.3 mM of palmitic acid (PA) with or without *Chlorella vulgaris* (CV) at 150 μg/mL, *Nannochloropsis gaditana* (NG) at 25 μg/mL and *Gracilaria vermiculophylla* (GV) at 150 μg/mL for 18 h. Data are means ± SEM (standard error of the mean). * *p* < 0.05 vs. control cells, # *p* < 0.05 vs. PA cells.

In PA cells, ALT/GPT level increased significantly compared to the control cells. Regarding those treated with the algae extracts, ALT/GPT levels were significantly reduced in *Chlorella vulgaris* and *Gracilaria vermiculophylla* cells when compared to PA cells. The concentration in the incubation medium of cells treated with *Nannochloropsis gaditana* was reduced (−37%), although statistical significance was not reached (Figure 4).

### 3.5. Effects on Genes and Proteins Involved in Triglyceride Metabolism

Gene and protein expressions were analysed in hepatocytes treated with the dose of each algae extract showing the greatest reduction in triglyceride content: that is, 150 μg/mL for *Chlorella vulgaris* and *Gracilaria vermiculophylla* and 25 μg/mL for *Nannochloropsis gaditana*. 

**Figure 5:**
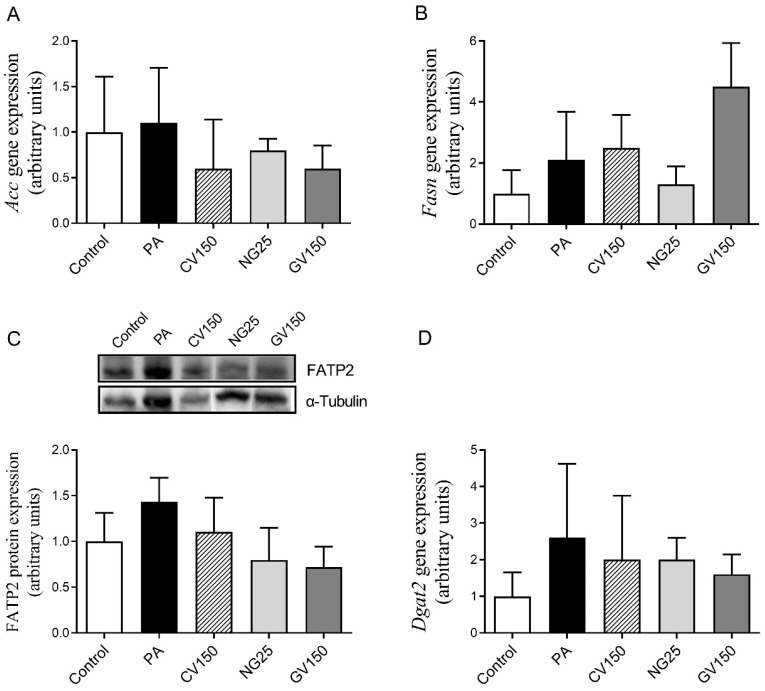
Gene expression of *Acc* (**A**) and *Fasn* (**B**), protein expression of FATP2 (**C**) and gene expression of *Dgat2* (**D**) in AML12 hepatocytes exposed or not to 0.3 mM of palmitic acid (PA) with or without *Chlorella vulgaris* (CV) at 150 μg/mL, *Nannochloropsis gaditana* (NG) at 25 μg/mL and *Gracilaria vermiculophylla* (GV) at 150 μg/mL for 18 h. Data are means ± SEM (standard error of the mean). The western blot bands shown are representative of 6 samples/group. *Acc*: acetyl-CoA carboxylase; *Dgat2*: diacylglycerol acyltransferase; *Fasn*: fatty acid synthase; FATP2: fatty acid transport protein 2.

Concerning the metabolic pathways that contribute to triglyceride accumulation in liver, the expression of genes and proteins involving fatty acid synthesis, fatty acid uptake and triglyceride assembly were analysed. Regarding de novo lipogenesis, gene expressions of *Acc* (Figure 5A) and *Fasn* (Figure 5B) were not significantly modified by any of the treatments. Gene expression of *Fatp2*, which is a transmembrane protein in charge of exogenous long chain fatty acid uptake, was not detected, and mRNA levels of *Dgat2*, involved in triglyceride assembly, remained unchanged (Figure 5D). In view of the fact that *Fatp2* gene expression was not detected, FATP2 protein expression was measured, and it was observed that it remained unchanged after the treatment with algae extracts. Nevertheless, it is worth mentioning that a non-statistically significant trend towards reduced levels (−50%) was appreciated in hepatocytes treated with *Gracilaria vermiculophylla* (*p* = 0.084) (Figure 5C) when compared to the PA group.

**Figure 6:**
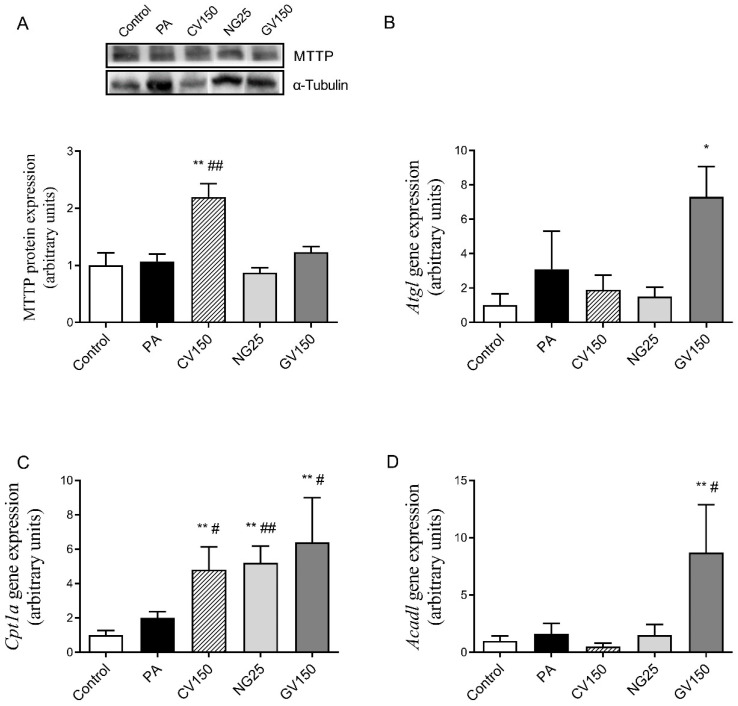
Protein expression of MTTP (**A**) and gene expression of *Atgl* (**B**), *Cpt1a* (**C**) and *Acadl* (**D**) in AML12 hepatocytes exposed or not to 0.3 mM of palmitic acid (PA) with or without *Chlorella vulgaris* (CV) at 150 μg/mL, *Nannochloropsis gaditana* (NG) at 25 μg/mL and *Gracilaria vermiculophylla* (GV) at 150 μg/mL for 18 h. Data are means ± SEM (standard error of the mean). * *p* < 0.05, ** *p* < 0.01 vs. control cells. # *p* < 0.05 and ## *p* < 0.01 vs. PA cells. *Acadl*: long chain acyl-CoA dehydrogenase; *Atgl*: adipose triglyceride lipase; *Cpt1a*: carnitine palmitoyltransferase 1a; MTTP: microsomal triglyceride transfer protein.

With regard to triglyceride secretion, only hepatocytes treated with *Chlorella vulgaris* showed a significant increase in MTTP protein expression; the rest of the algae treatments induced no changes (Figure 6A). Gene expression of *Atgl*, which is responsible for triglyceride hydrolysis, was not modified by any of the algae treatments when compared with PA incubation alone, although its expression was increased by 133% in the GV group (Figure 6B). Regarding fatty acid oxidation, gene expression of *Cpt1a* and *Acadl* were analysed; the first one is responsible for the transportation of long-chain fatty acids into mitochondrial matrix for beta-oxidation, and hile the second gene catalyses the first step of mitochondrial fatty acid beta-oxidation. *Cpt1a* showed a significant increase in cells treated with the three algae extracts (Figure 6C). Lastly, only *Gracilaria vermiculophylla* extract increased mRNA levels of *Acadl* (Figure 6D). 

**Figure 7:**
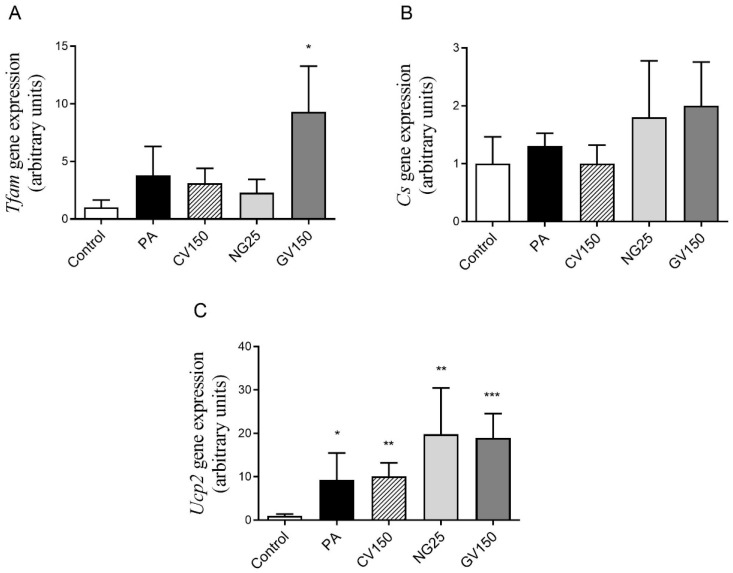
Gene expression of *Tfam* (**A**) and *Cs* (**B**) and *Ucp2* (**C**) in AML12 hepatocytes exposed or not to 0.3 mM of palmitic acid (PA) with or without *Chlorella vulgaris* (CV) at 150 μg/mL, *Nannochloropsis gaditana* (NG) at 25 μg/mL and *Gracilaria vermiculophylla* (GV) at 150 μg/mL for 18 h. Data are means ± SEM (standard error of the mean).* *p* < 0.05, ** *p* < 0.01 and *** *p* < 0.001 vs. control group. *Cs*: citrate synthase; *Tfam*: mitochondrial transcription factor A, *Ucp2*: uncoupling protein 2.

mRNA levels of *Cs* and *Tfam*, related to mitochondrial density and mitochondriogenesis, respectively, were not modified by algae treatments in comparison with cells incubated only with PA. However, it is worth mentioning that an increase of 145% was observed in the GV group. Concerning *Ucp2*, a possible indicator of NAFLD development, the treatment with algae extracts did not modify its expression in comparison with the PA group (Figure 7).

## 4. Discussion

The increasing prevalence of NAFLD has encouraged the interest in new strategies for its prevention and treatment. In this scenario, microalgae and macroalgae extracts have received great attention from the scientific community in recent years. Data from the literature show that, in addition to the effects attributed to the pigments present in algae, some extracts containing high percentages of protein-like compounds and carbohydrates have been reported to be able to modulate lipid metabolism [23,24,25,26,27,28,29]. In the present study, the efficacy on liver steatosis of extracts rich in proteins and peptides, obtained from the microalgae *Chlorella vulgaris* and *Nannochloropsis gaditana* and the macroalga *Gracilaria vermiculophylla*, was assessed.

Regarding algae extract composition, in that obtained from *Chlorella vulgaris*, the most intense protein band was detected within the range of 20–25 kDa. In this line, a previous study showed that the main band for the proteins extracted from *Chlorella vulgaris* at alkaline pH was located at 30 kDa. However, an intense band corresponding to proteins of more than 670 kDa and 50–75 kDa was also observed [30]. In another study carried out in a different species of *Chlorella*, protein extracts produced by alkaline solubilisation corresponded to ~8 kDa [31]. In the extract obtained from *Nannochloropsis gaditana*, the main protein bands corresponded to 37 kDa, 50 kDa and 100 kDa. In this case, only the band at 50 kDa had been previously detected for this microalga species [32]. In the study reported by Vizcaíno et al. [32], molecular weight bands lower than 14 kDa were also visualised in the native microalga, but not in the present study. Moreover, in a piece of research addressed with another species of *Nannochloropsis*, proteins extracted by alkaline solubilisation showed major peaks corresponding to 10 kDa and 1.6 kDa [31]. Regarding the macroalga *Gracilaria vermiculophylla*, it seems that the proteins present in the extract had very limited solubility, probably due to the formation of protein–polysaccharide complexes, thus hindering their characterisation by SDS-PAGE and HPLC-SEC. Interestingly, in the case of the *Chlorella vulgaris*, low molecular weight peptides may be interacting with pigments. The differences observed between the results obtained in the present study and those reported in the literature can be due to the extraction protocol applied. A limitation of the present study is that the proteins and peptides present in the extracts have not been identified. Thus, a more precise characterisation is an interesting aspect to be addressed in future studies.

In the present study, the concentrations of the extracts utilised for hepatocyte incubation ranged from 10 μg/mL to 150 μg/mL, a range of doses commonly used with algae extracts. In this experiment, palmitic acid was used to stimulate triglyceride accumulation into the cells; and to mimic the influx of excess free fatty acids into hepatocytes, which takes place under overfeeding conditions and obesity, and thus, to induce steatosis. Under the present experimental conditions, the data demonstrate that the three algae extracts partially prevented the accumulation of triglycerides induced by palmitic acid in cells; the percentages of prevention ranged from 18% to 34%. The microalga *Nannochloropsis gaditana* was the most effective because it induced the highest reduction in triglyceride accumulation (−34%); it was also the most powerful since it was effective at a dose lower than the other algae (10 μg/mL). This fact could be due to their high content in soluble proteins and peptides. In the case of this microalga, surprisingly, whereas 10 and 25 μg/mL were effective, higher doses, 50 and 150 μg/mL, were not. This phenomenon has also been observed in studies devoted to analysing the metabolic effects of phytochemicals such as phenolic compounds, where on occasion, a lower dose appears to be more effective than a higher one [18,33,34]. Nevertheless, the reasons that justify this phenomenon have not been described to date. In contrast, the *Gracilaria vermiculophylla* extract showed a dose response pattern on triglyceride accumulation. 

These beneficial effects are probably caused by the proteins and peptides present in the extracts. Moreover, in the case of *Nannochloropsis gaditana*, it has been observed that its protein and peptides are biologically active [26]. In fact, it has been recently discovered that it contains UCA01, a protein of around 25 and 37 KDa with anti-tumoral, antioxidant and mitochondrial protective effects on HepG2 hepatocytes (patent number: 201930775) [35,36].

Although algae have been recognised and exploited as alternative food supplements, it has been reported that some species can produce compounds with toxic effects [6,37]. In order to address this issue, hepatocyte viability was assessed, and no significant effects were observed for any algae at any of the doses tested. Moreover, a decrease in ALT levels was found when cells were treated with *Chlorella vulgaris* and *Gracilaria vermiculophylla*, indicating a protective effect on hepatocytes. In the case of the *Nannochloropsis gaditana* extract, no differences on its secretion were identified between this group and the PA group.

After confirming the positive effects of the algae extracts, the potential mechanisms underlying the anti-steatotic effects were assessed. For this purpose, the sets of cells treated with the dose of each algae extract showing the greatest reduction in triglyceride content were used (*Chlorella vulgaris* at 150 μg/mL, *Nannochloropsis gaditana* at 25 μg/mL and *Gracilaria vermiculophylla* at 150 μg/mL.) The genes and proteins analysed are related to the main metabolic pathways involved in hepatic triglyceride accumulation: (a) pathways favouring triglyceride accumulation, de novo lipogenesis and fatty acid uptake for the synthesis and triglyceride assembly, and (b) pathways avoiding triglyceride accumulation, triglyceride mobilization, fatty acid oxidation and triglyceride secretion. 

In the case of *Chlorella vulgaris*, the results suggest that one mechanism involved in the prevention of triglyceride accumulation intensified triglyceride secretion from hepatocytes, mediated by a boost in MTTP protein expression. *Cpt1a* was also increased, suggesting enhanced fatty acid oxidation, and thus, a lower availability of free fatty acids for triglyceride synthesis. According to data concerning *Cpt1a* gene expression, the triglyceride-lowering effect of *Nannochloropsis gaditana* was probably caused by an increase in fatty acid oxidation. In the case of *Gracilaria vermiculophylla*, the results show a trend towards reduced fatty acid uptake (FATP2) and an augmented lipolysis (*Atgl*) that can be linked to the increase in fatty acid oxidation (*Cpt1a, Acadl* and *Tfam*). 

Due to the fact that a great number of compounds with potential anti-steatotic effect are present in the three algae extracts, it is not possible to state which of them is truly responsible for the positive effects induced by the algae in hepatocytes. However, the identification of the bioactive compounds or metabolites from algae extracts that induce the beneficial effects will be crucial in the upcoming years for the development of new therapies targeting fatty liver and other metabolic diseases; thus, it requires further research.

In conclusion, the present study demonstrates for the first time that *Chlorella vulgaris*, *Nannochloropsis gaditana* and *Gracilaria vermiculophylla* extracts abundant in proteins are able to partially prevent the accumulation of triglycerides induced by palmitic acid in cultured hepatocytes, a model used to mimic the steatosis induced in liver by dietary patterns rich in saturated fat. The three algae extracts analysed prevent hepatic steatosis by acting on different metabolic pathways involved in hepatic lipid metabolism. Nevertheless, further research is needed to validate these results in in vivo studies and to test the effects of these extracts on other processes involved in the development of liver steatosis and its progression to steatohepatitis, such as oxidative stress and inflammation. 

## Figures and Tables

**Table 1:** Primer sequences, gene accession and assay ID for quantitative Real-Time PCR amplification.

**SYBR Green RT-PCR**			
**Gene**	**Gene Accession**	**Sense Primer 5′-3′**	**Antisense Primer 5′-3′**
*Acadl*	NM_007381.4	TGG GGA CTT GCT CTC AAC A	GGC CTG TGC AAT TGG AGT
*Actb*	NM_007393.5	ACG AGG CCC AGA GCA AGA G	GGT GTG GTG CCA GAT CTT CTC
*Atgl*	NM_025802.3	GAG CTT CGC GTC ACC AAC	CAC ATC TCT CGG AGG ACC A
*Cpt1a*	NM_013495.2	CGG TTC AAG AAT GGC ATC ATC	TCA CAC CCA CCA CCA CGA T
*Cs*	NM_026444.4	GCC TCT GCA TGG ACT AGC AAA	TTG CCG ACT TCC TTC TGT AGC T
*Fasn*	NM_007988.3	AGC CCC TCA AGT GCA CAG T	TGC CAA TGT GTT TTC CCT G
*Tfam*	NM_009360.4	AAG CTT ATC CAT GAC AGC TAA AGG	GGC TGG CTC ACC ACA GTT
*Ucp2*	NM_011671.5	TAC TCT CCT GAA AGC CAA CCT C	CAA TGA CGG TGG TGC AGA AG
**Taqman RT-PCR**			
**Gene**	**Gene Accession**	**Assay ID**	
*Acc*	NM_133360.2	Mm01304285_m1	
*Actb*	NM_007393.5	Mm02619580_g1	
*Dgat2*	NM_026384.3	Mm00499536_m1	

*Acadl*: long chain acyl-CoA dehydrogenase; *Acc*: acetyl-CoA carboxylase; *Actb*: beta actin *Atgl*: *adipose triglyceride lipase*; *Cpt1a*: carnitine palmitoyltransferase I A, *Cs*: citrate *synthase*; *Dgat2*: diacylglycerol acyltransferase; *Fasn*: fatty acid synthase; *Tfam*: mitochondrial transcription factor A; *Ucp2*: uncoupling protein 2.

**Table 2:** Proximal composition of the algae extracts used in the present study.

	Protein (%)	Fat (%)	Ash (%)	Carbohydrates (%)
*Chlorella vulgaris*	48.0 ± 0.2	11.8 ± 2.0	20.5 ± 0.6	21.1 ± 0.6
*Nannochloropsis gaditana*	42.0 ± 9.2	5.6 ± 0.1	42.6 ± 4.2	9.9 ± 4.4
*Gracilaria vermiculophylla*	41.4 ± 0.7	3.5 ± 0.1	22.1 ± 3.2	33.4 ± 3.6

% of dry weight (DW). Carbohydrates were calculated by difference.

## Data Availability

Not applicable.
